# Massive iatrogenic duodenal deficiency: originating from and resolved by endoscopic treatment

**DOI:** 10.1055/a-2842-0618

**Published:** 2026-04-20

**Authors:** Fan Wang, Shouquan Dong, Rui Zhou, Feng Zhou, Qiu Zhao, Hongling Wang

**Affiliations:** 1 89674 Department of Gastroenterology, Zhongnan Hospital of Wuhan University, Wuhan, China

**Fig. 1:**
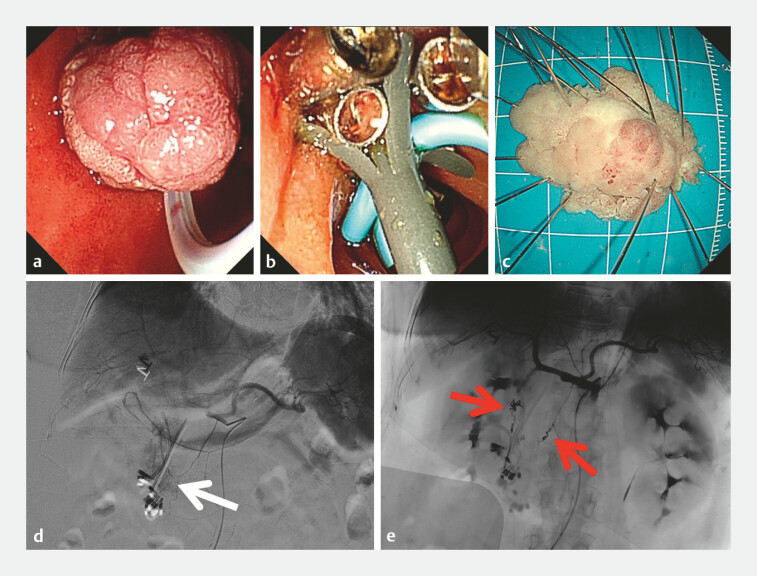
**a–c** Endoscopic resection of duodenal papilla adenoma, wound suturing and placement of biliary and pancreatic stents on day 0. **d, e** Gastroduodenal artery and ectopic pancreaticoduodenal artery with signs of contrast extravasation at the distal end (white arrow), and interventional embolization (red arrow) at the same day.

**Fig. 2:**
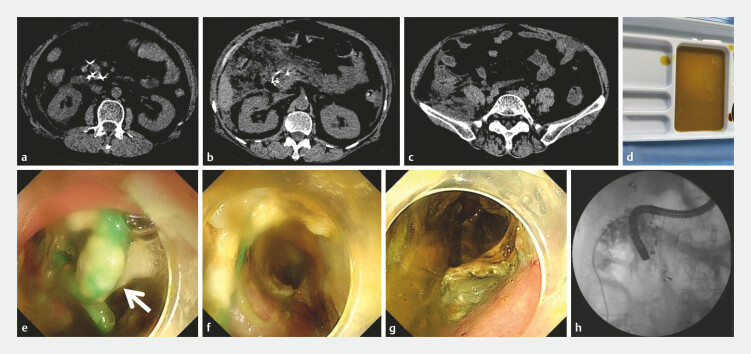
**a** CT scan indication of no obvious pancreatitis, perforation or abscess formation on day 2. **b, c** Repeat CT scan indication of retroperitoneal abscess from the periduodenum to pelvic cavity on day 13. **d** Percutaneous puncture drainage of pus and digestive fluid on day 14. **e–h** Endoscopic indication of the duodenal perforation and a retroperitoneal abscess on day 28 (arrow: the tip of the percutaneous drainage tube). CT, computed tomography.

A 63-year-old woman with a 10-year history of systemic lupus erythematosus (SLE) managed with glucocorticoids and immunosuppressants was admitted for a villous tubular adenoma of the duodenal papilla. Endoscopic resection of the mass was performed ( Fig. 1 ). Five hours after the procedure, the patient developed severe hematemesis, which was managed with interventional embolization ( Video 1 ). Post-procedural hyperamylasemia was observed, which normalized following a 3-day course of somatostatin therapy. The peak serum amylase level recorded was 1559 U/L (reference range: 35~135 U/L). Subsequently, the patient experienced intermittent fever and abdominal pain. On day 14, a repeat computed tomographic scan revealed extensive periduodenal exudate and gas accumulation ( Fig. 2 ). Percutaneous puncture drainage yielded pus and digestive fluid.

Endoscopic treatment of massive iatrogenic duodenal deficiency.Video 1

On day 28, gastroscopy revealed the tip of the percutaneous drainage tube, suppuration and a lack of integrity in the wall in of the descending duodenum, confirming the duodenal perforation with a retroperitoneal abscess. A nasojejunal tube was placed into the jejunum for enteral nutrition. The multidisciplinary team recommended optimizing drainage and nutritional support to control the infection and improve the patient’s general condition prior to an elective pancreaticoduodenectomy. However, despite these intensified measures, the infection worsened.

**Fig. 3:**
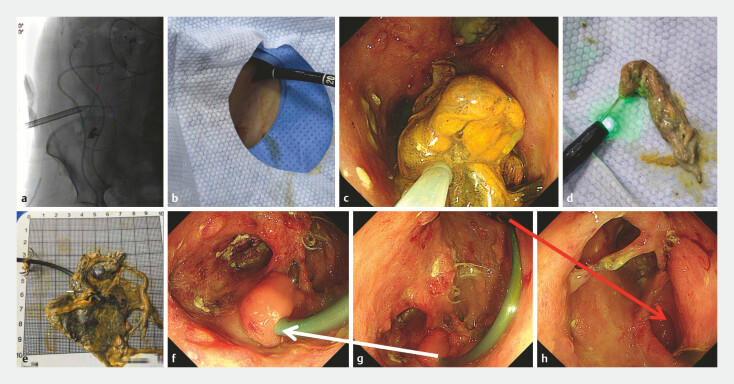
**a–d** Endoscopic debridement of the retroperitoneal necrosis through the percutaneous sinus tract on day 38. **e–h** Debridement of the entire necrotic descending duodenum, and complete separation of the duodenal bulb (white arrow) from the horizontal duodenum (red arrow).

**Fig. 4:**
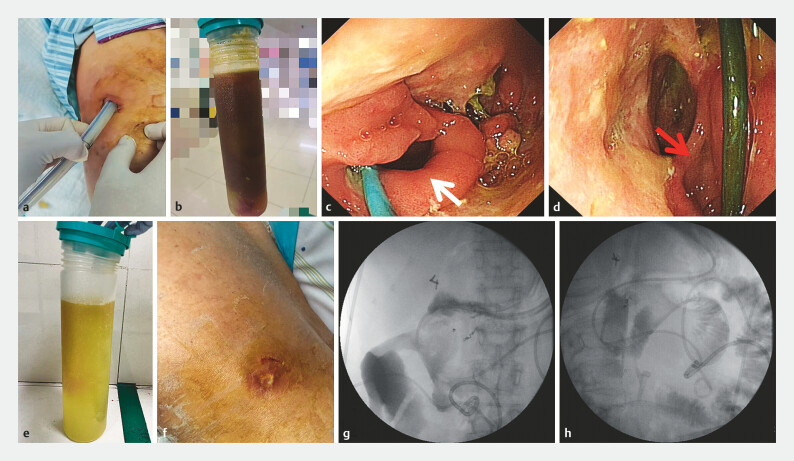
**a, b** Continuous massive saline lavage through the percutaneous tube (30-French) and cloudy drainage fluid. **c–e** Smooth walls of the retroperitoneal abscess (white arrow: duodenal bulb and red arrow: horizontal duodenum) and clear drainage fluid on day 63. **f** Percutaneous sinus closure after removing the drainage tube on day 76. **g, h** An oral contrast agent localized to the retroperitoneal cavity and smoothly flowed through the cavity into the jejunum on day 77.

On day 38, endoscopic debridement was performed via the percutaneous sinus tract. Massive necrosis, including the entire descending duodenum, was cleared from the purulent cavity ( Fig. 3 ). The duodenal bulb was found to be completely separated from the horizontal duodenum. Subsequent continuous massive saline lavage and one additional endoscopic debridement significantly reduced the size of the cavity and smoothed its wall ( Fig. 4 ). The patient’s condition improved markedly. Transient hyperbilirubinaemia was observed, which normalized after 2-week treatment of ademetionine. The peak serum total bilirubin level recorded was 132.5 μmol/L (reference range: ≤23 μmol/L).

**Fig. 5:**
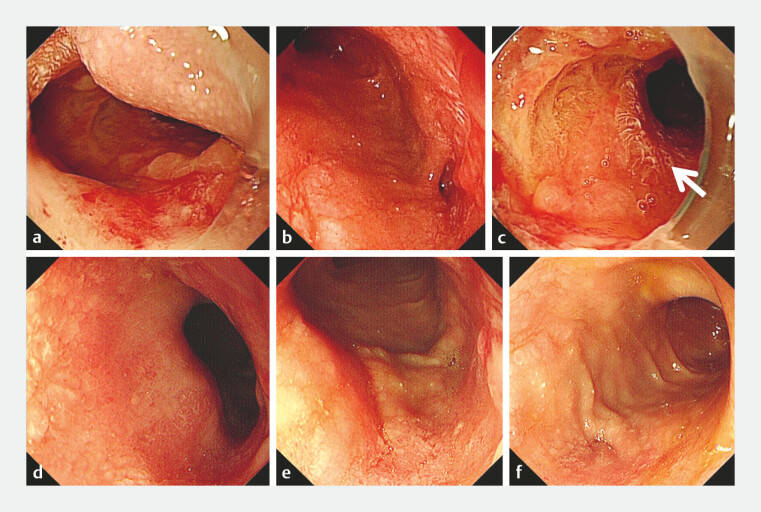
One-month post-discharge gastroscopy indication of mild stenosis in the duodenal bulb ( **a** ), a formed luminal structure between the bulb and the horizontal duodenum ( **b** ), and evidence of mucosal growth extending from the horizontal part to the bulb (arrow) ( **c** ). **d–f** Three-month post-discharge gastroscopy indication of milder inflammation.

By day 76, the percutaneous sinus had closed after the removal of the drainage tube and maintenance of nasal negative pressure drainage. An oral contrast agent examination showed that contrast flowed smoothly from the duodenal bulb through the former cavity and into the jejunum. On day 88, the patient was discharged on an oral liquid diet. One month later, the patient was able to tolerate solid food without discomfort. Follow-up gastroscopy revealed mild stenosis in the duodenal bulb, a formed luminal structure between the bulb and the horizontal duodenum, and evidence of mucosal growth extending from the horizontal part toward the bulb ( Fig. 5 ).

Duodenal deficiency represent a complex clinical challenge with high mortality and often mandate surgical intervention 1 2 . However, the optimal treatment strategy must be tailored to the individual patient’s condition 3 . Valuable insights were gained from this case. First, caution is warranted when considering interventional embolization for endoscopic-related duodenal hemorrhage, particularly in patients with comorbidities that may affect vascular integrity. In this case, the patient’s SLE and the angiographic finding of slender vessels placed her at high risk of duodenal ischemic necrosis following embolization. Second, this case underscores the remarkable regenerative capacity of the human body. With adequate nutritional support and infection control, the localized retroperitoneal cavity was able to serve as a conduit for enteric contents, effectively functioning as the missing intestinal lumen, while the remaining duodenum underwent spontaneous repair.

In conclusion, this rare case of massive iatrogenic duodenal deficiency, which both originated from and was ultimately resolved by endoscopic treatment, provides a critical reminder of a serious potential complication for endoscopists and a novel minimally invasive alternative for poor surgical candidates.

Endoscopy_UCTN_Code_TTT_1AO_2AI
